# Spermidine enhanced resistance of *Chlorella* to high levels of CO_2_ and light intensity for improving photosynthetic growth rate

**DOI:** 10.1039/c9ra05152j

**Published:** 2019-08-23

**Authors:** Xiangdong Zhang, Jun Cheng, Hongxiang Lu, Feifei Chu, Junchen Xu, Xuebin Wang, Kefa Cen

**Affiliations:** State Key Laboratory of Clean Energy Utilization, Zhejiang University Hangzhou 310027 China juncheng@zju.edu.cn +86 571 87951616 +86 571 87952889; College of Standardization, China Jiliang University Hangzhou 310018 China; Penglai Power Generation Company Ltd. of China Energy Investment Corporation Penglai 265601 China

## Abstract

In order to promote the photosynthetic growth rate of *Chlorella* in the presence of flue gas CO_2_ from coal-fired power plants, spermidine was first used to enhance cellular resistance to a high CO_2_ concentration (15%) and high light intensity (30 000 lux). It was found that low concentrations (100–300 μM) of spermidine significantly enhanced the photosynthetic growth rate of *Chlorella*. The accelerated cell division decreased the cell diameter from 3.64 μm to 2.71 μm and the fractal dimension from 1.60 to 1.49, and the activity of total superoxide dismutase (T-SOD) increased from 0.48 U mL^−1^ to 5.33 U mL^−1^. Expression levels of key enzymes of photosystems I and II, ATP synthase and transportase markedly increased, thereby enhancing the electron transport and energy supply that reduced oxidative damage. Finally, an enhanced cellular resistance to the high CO_2_ concentration and high light intensity increased the biomass yield from 0.11 g L^−1^ to 1.71 g L^−1^ (300 μM).

## Abstract 2

Spermidine enhanced resistance of *Chlorella* to high levels of CO_2_ and light intensity.

## Graphical abstract

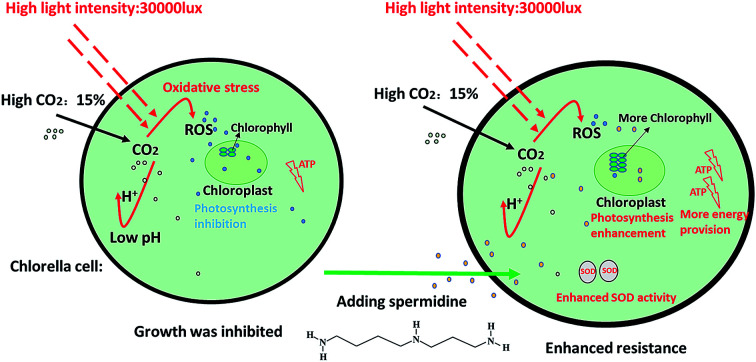

## 1. Introduction

Polyamines are required for optimal growth in most cells. They are involved in a variety of cellular processes, such as gene expression, cell growth, survival, stress response and proliferation.^1^ Polyamines can indirectly regulate the expression of related genes during stress response. In plants, exogenous administration of various concentrations of putrescine, spermidine and spermine was shown to confer enhanced tolerance to various stresses.^2^ Zhang *et al.*^3^ found that spermidine efficiently alleviated the inhibitory effects of saline–alkaline stress on plant growth and inhibited saline–alkaline stress-induced H_2_O_2_ and O^2−^ accumulation. Lou *et al.*^4^ found that exogenous application of 20 μM spermidine effectively alleviated salt-induced damage in *Medicago sativa* (alfalfa). Murkowski^5^ found exogenous spermidine alleviated salinity–alkalinity stress damage using antioxidant enzymes and non-enzymatic systems in chloroplasts. Additionally, exogenous spermidine supplementation was confirmed to alleviate salt stress in sorghum (*Sorghum bicolor*) seedlings, as well as the growth inhibition and damage to the structure and function of the photosynthetic apparatus caused by drought stress in *Phyllostachys edulis* seedlings.^6–8^

Reports on polyamine-induced stress resistance and promotion of microalgal growth are currently insufficient. Czerpak *et al.*^9^ studied the growth and cellular contents of chlorophyll a and b, monosaccharides and proteins in the alga *Chlorella vulgaris* Beijerinck (Chlorophyceae) under different concentrations of agmatine, putrescine, spermidine and spermine. However, this study did not cover in-depth the stress resistance of microalgae or their reaction mechanisms, nor did it investigate the effects on photosynthesis-related proteins, fluorescence parameters, cell microstructure or SOD. Piotrowska-Niczyporuk *et al.*^10^ studied the green microalga *Chlorella vulgaris* (Chlorophyceae) exposed to heavy metal (Cd, Cu, Pb) stress and found that exogenous application of polyamine–spermidine alleviated stress symptoms by inhibiting heavy metal biosorption, while restoring algal growth and primary metabolite levels. Kim *et al.*^11^ confirmed that high spermidine levels helped engineered *Saccharomyces cerevisiae* strains to resist the toxicity of chemicals, such as acetic acid and furfural, thereby mitigating the effects of acid stress. Thus, it is very crucial to study spermidine-induced stress resistance and reaction mechanisms in microalgae.

At present, power plant flue gas is widely used for culturing the microalga *Chlorella*. There have been many studies on *Chlorella* cultivation using actual power plant flue gas and artificial simulated flue gas^12,13^ because a high concentration of 15% CO_2_ results in decreased culture solution pH, which is detrimental to cell growth. Currently, raceway ponds are mainly used for large-scale cultivation of microalgae.^14,15^ Compared with laboratory conditions, the microalgal concentration in industrial raceway ponds is low. Furthermore, these microalgae are often exposed to adverse environmental conditions, such as high light intensity and low pH, which inhibits their growth. Polyamines play an important role in regulating biological growth and stress resistance under adverse conditions, including high temperature, salt stress and high light intensity. To the best of our knowledge, there is no literature on the effects of polyamines on microalgal growth under high CO_2_ concentrations and high light intensity, nor is there relevant research on cell surface morphology and microstructure under these conditions. The most common polyamines are putrescine, spermidine and spermine. Most studies on various stress resistances have also focused on the above three polyamines. It has been suggested that spermidine is the most efficient of the three major polyamines (putrescine, spermidine, spermine) at restoring maximum photochemical efficiency (*F*_v_/*F*_m_) to low-salt-stressed thylakoids.^16^ It was also found that exogenous application of spermidine to *Physcia semipinnata* resulted in higher chlorophyll a content and PSII activity than application of spermine or putrescine in plants exposed to UV-A radiation.^17^ Thus, this paper focused on the study of spermidine in view of high light intensity and high concentration of CO_2_. It is of great practical significance to study the effects of different spermidine concentrations on the stress resistance of *Chlorella* in an actual industrial environment.

The objective of this research was to promote the growth rate of *Chlorella* by enhancing spermidine-induced resistance to a high CO_2_ concentration (15%) and high light intensity (30 000 lux). Photosynthesis-related proteins, fluorescence parameters, cell microstructure and SOD activity of *Chlorella* were studied in-depth to understand how spermidine improved the microalgal growth rate and stress resistance. Lastly, the molecular mechanism of spermidine-induced stress resistance of *Chlorella* was revealed. This research will help improve microalgal cultivation in tubular or raceway pond reactors that are used to treat high concentrations of CO_2_ from power plant flue gas.

## 2. Materials and methods

### 2.1. Microalgae and culture medium

The microalgal strain used in this study was *Chlorella* sp. MS700. Optimized f/2 artificial seawater medium,^18^ used for microalgal cultivation, was composed of (a) macro elements NaCl (21.194 g L^−1^), Na_2_SO_4_ (3.55 g L^−1^), KCl (0.599 g L^−1^), CaCl_2_ (1.015 g L^−1^), KBr (0.0863 g L^−1^), H_3_BO_3_ (0.0230 g L^−1^), NaF (0.0028 g L^−1^), MgCl_2_·6H_2_O (9.592 g L^−1^), SrCl_2_·6H_2_O (0.0218 g L^−1^); (b) enriched nutrients NaNO_3_ (0.225 g L^−1^), NaH_2_PO_4_·H_2_O (0.025 g L^−1^); (c) trace metal solution (1 mL L^−1^); and (d) vitamin solution (1 mL L^−1^). The trace metal solution contained FeCl_3_·6H_2_O (3.15 g L^−1^), Na_2_EDTA·2H_2_O (4.36 g L^−1^), MnCl_2_·4H_2_O (0.180 g L^−1^), ZnSO_4_·7H_2_O (0.022 g L^−1^), CoCl_2_·6H_2_O (0.010 g L^−1^), CuSO_4_·5H_2_O (0.0098 g L^−1^), Na_2_MoO_4_·2H_2_O (0.0063 g L^−1^) and NiCl_2_·6H_2_O (0.00149 g L^−1^). The vitamin solution contained vitamin B_1_ (200 g L^−1^), vitamin H (1 mg L^−1^) and vitamin B_12_ (1 mg L^−1^). The medium was sterilized in an autoclave for 30 min at 121 °C (Boxun YXQ-LS-75811, Shanghai, China). The vitamin solution was added into the culture medium on a clean bench after the medium was autoclaved; the salinity was 3%.

The spermidine [*N*-(-3-aminopropyl)-1,4-diaminobutane] used in the experiment was purchased from Macklin (CAS: 124-20-9; purity: 99%). Stock solution (10^−3^ M) was prepared in H_2_O and aliquots were stored at −20 °C. In the subsequent experiments, a spermidine gradient was set according to the desired treatment.

### 2.2. Experimental conditions and biomass measurements

The algae were cultivated at 27 °C in a 600 mL bioreactor (183 mm height, 76 mm inner diameter) in an artificial greenhouse. The light intensity was controlled at 30 000 lux. A mixture of 15% CO_2_/85% N_2_ was introduced into the bioreactor at a flow rate of 60 mL min^−1^ using a flowmeter (Seven-star CS200, China).

The experimental process was divided into two stages. Phase I (days 0–5) was the growth inhibition phase, in which no spermidine was added. Phase II (days 6–14) was the treatment phase. Different volumes of spermidine stock solution were added at the end of day 5. The spermidine concentrations of the treatments were 0 (control), 10, 30, 100, 200, 300 and 400 μM. Additionally, a 100 μM NaOH treatment was set up. Each treatment was performed in duplicate. The OD_680_ of the microalgal samples were measured with a UV/visible spectrophotometer (Unico UV2600, USA) every 24 h during cultivation. The microalgal samples were diluted to ensure the absorbance reading was lower than 1.0. At the end of cultivation (day 14), 10 mL of sample was dewatered by centrifugation (Beckman Avanti J26-XP, USA) at 7500 rpm for 7 min and then washed three times with deionized water. Finally, the microalgal pellet was collected. The dry weight was measured after drying the microalgal pellet at 105 °C for 24 h.

The relationship between the dry weight and absorbance of the microalgal biomass was established as follows (1):

1 Biomass dry weight (g L^−1^) = 0.2584 × OD_680_, *r*^2^ = 0.9962

The dynamic growth rate of the microalgae was calculated as shown in the following eqn (2):2
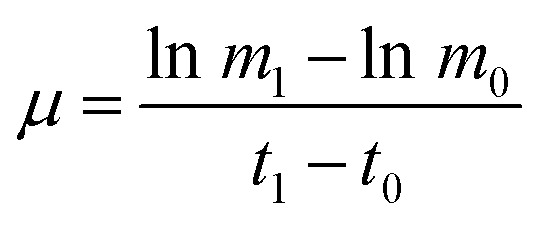
where *m*_1_ is the dry weight at time *t*_1_ and *m*_0_ is the dry weight at time *t*_0_.

### 2.3. Transmission electron microscopy and scanning electron microscopy

On day 10 of the experiment, 1 mL of algal solution under each treatment (0, 10, 30, 100, 300 μM) was centrifuged (6000 rpm, 5 min). The supernatant was discarded and then the pellet was stored overnight in 2.5% glutaraldehyde solution at 4 °C.

The microstructure of the samples was observed by transmission electron microscopy (TEM; H-7650, Hitachi, Japan). From the TEM images, dozens of cells were randomly selected and then analyzed with Nano Measurer software (Nano Measurer v1.2.5) to determine their cell diameter and cell wall thickness.

The cell surface microstructure of the samples was observed by scanning electron microscopy (SEM; SU-8010, Hitachi, Japan). Several SEM images at different magnifications were obtained and binarized using MATLAB software (MATLAB R2017b). Fractal Fox software was used to analyze the fractal dimension of the microalgal cells. The fractal dimension of *Chlorella* cells reflected the smoothness of the cell surface.

### 2.4. Measurement of T-SOD activity in microalgal cells

During the growth period, 30 mL of algal solution from each treatment (0, 30, 100, 300 μM) was taken every other day and then centrifugated (8000 rpm, 15 min, 4 °C) to collect the microalgal cells. The microalgal cells were resuspended in 0.05 M phosphate buffer (pH 7.8), which was precooled in an ice bath. After treatment for 30 minutes in an ice bath that was placed under an ultrasonic wave (5 W output), the solution was centrifuged at 12 000 rpm for 10 minutes. The supernatant was then used for analysis of T-SOD activity *via* the WST-1 method.

The Superoxide Dismutase Detection Kit (A001; Nanjing Jiancheng Bioengineering Institute, Nanjing, China) was selected for SOD measurement. One unit of SOD activity is defined as the amount of SOD corresponding to the SOD inhibition rate of 50% per mL of the reaction solution. The T-SOD was represented as follows (3):3
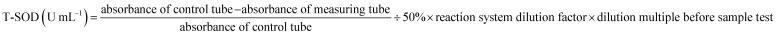


### 2.5. Analysis of chlorophyll content in microalgal cells

Algal solution (5 mL) was filtered every other day. Methanol (4 mL; purity >99.9%) was added to the algal pellet and placed in a dark environment for 30 minutes. The solution was then centrifuged at 6000 rpm for 5 minutes and then the absorbance of the supernatant was measured. The pigment concentration was calculated based on the method of Porra *et al.*^19^ Briefly, the absorbance of the extracted supernatant was measured spectrophotometrically at 652 nm (*A*_652_) and 665 nm (*A*_665_). Intracellular chlorophyll a and b concentrations were calculated using the following eqn (4) and (5):

4 Chlorophyll a (μg mL^−1^) = 16.29 × *A*_665_ − 8.54 × *A*_652_

5 Chlorophyll b (μg mL^−1^) = 30.66 × *A*_652_ − 13.58 × *A*_665_

### 2.6. Photosynthetic fluorescence parameter measurement

Chlorophyll fluorescence parameters of the microalgal cells were tested by pulse modulation fluorometer (FMS-2, Hansatech, Britain). The dark adaptation time in this experiment was 10 minutes. *F*_v_/*F*_m_ was calculated as eqn (6):^20^

6 *F*_v_/*F*_m_ = (*F*_m_ − *F*_o_)/*F*_m_

where *F*_o_ represents the minimum fluorescence parameter (the fluorescence intensity of the fully dark-adapted photosynthetic mechanism when all photosystem II (PSII) reaction centers are open); and *F*_m_ represents the maximum fluorescence (the fluorescence intensity of the fully dark-adapted photosynthetic mechanism when all PSII reaction centers are closed).

### 2.7. Transcriptomic analysis

On day 10, two experimental treatments (0 μM and 100 μM) were selected to harvest a sufficient amount of algae (dry algae powder > 1 g). The microalgal cells were collected by high-speed refrigerated centrifugation, quick-frozen in liquid nitrogen, collected and then stored at −80 °C for further analysis. The clustering of the index-coded samples was performed on a cBot Cluster Generation System using TruSeq PE Cluster Kit v3-cBot-HS (Illumina) and subsequent analysis was completed as per Pei *et al.*^21^

## 3. Results and discussion

### 3.1. Effect of different concentrations of spermidine on *Chlorella* growth

**Fig. 1:**
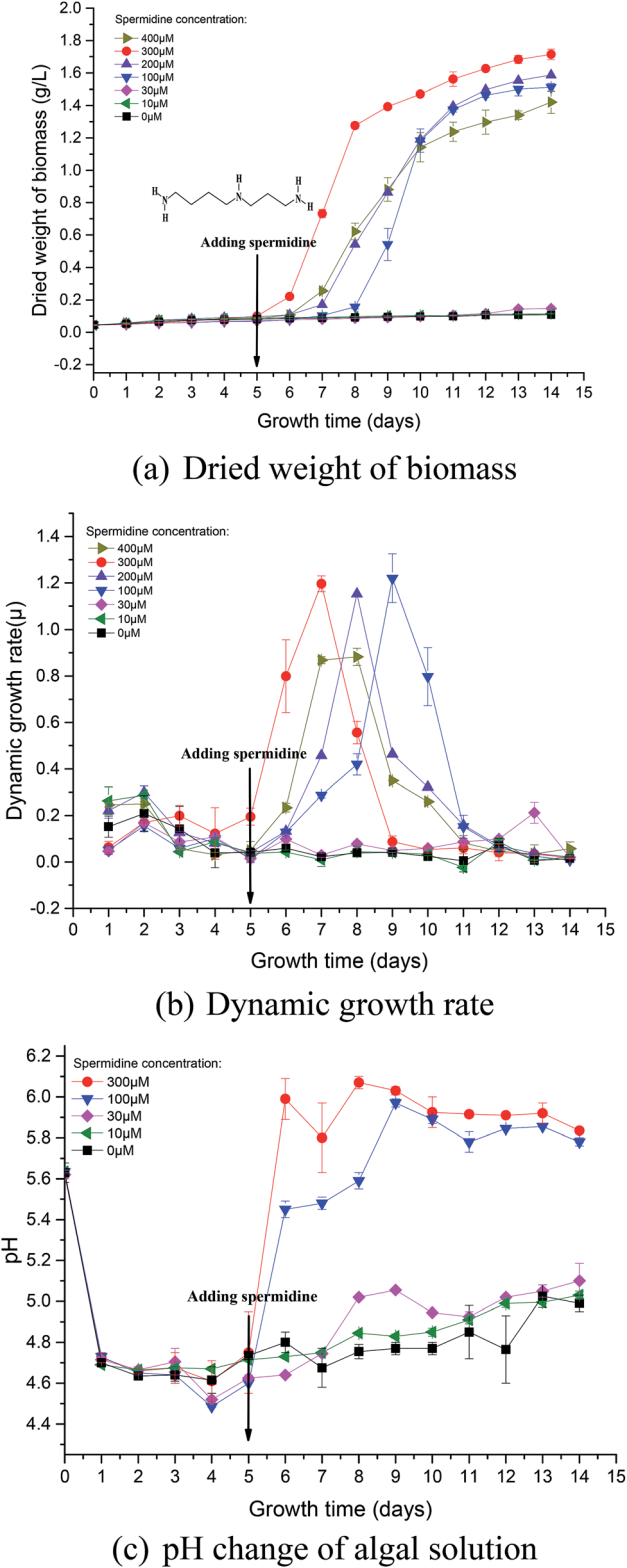
Growth curves (a and b) and pH change (c) of *Chlorella* under a high CO_2_ concentration (15%) and high light intensity (30 000 lux).

**Fig. 2:**
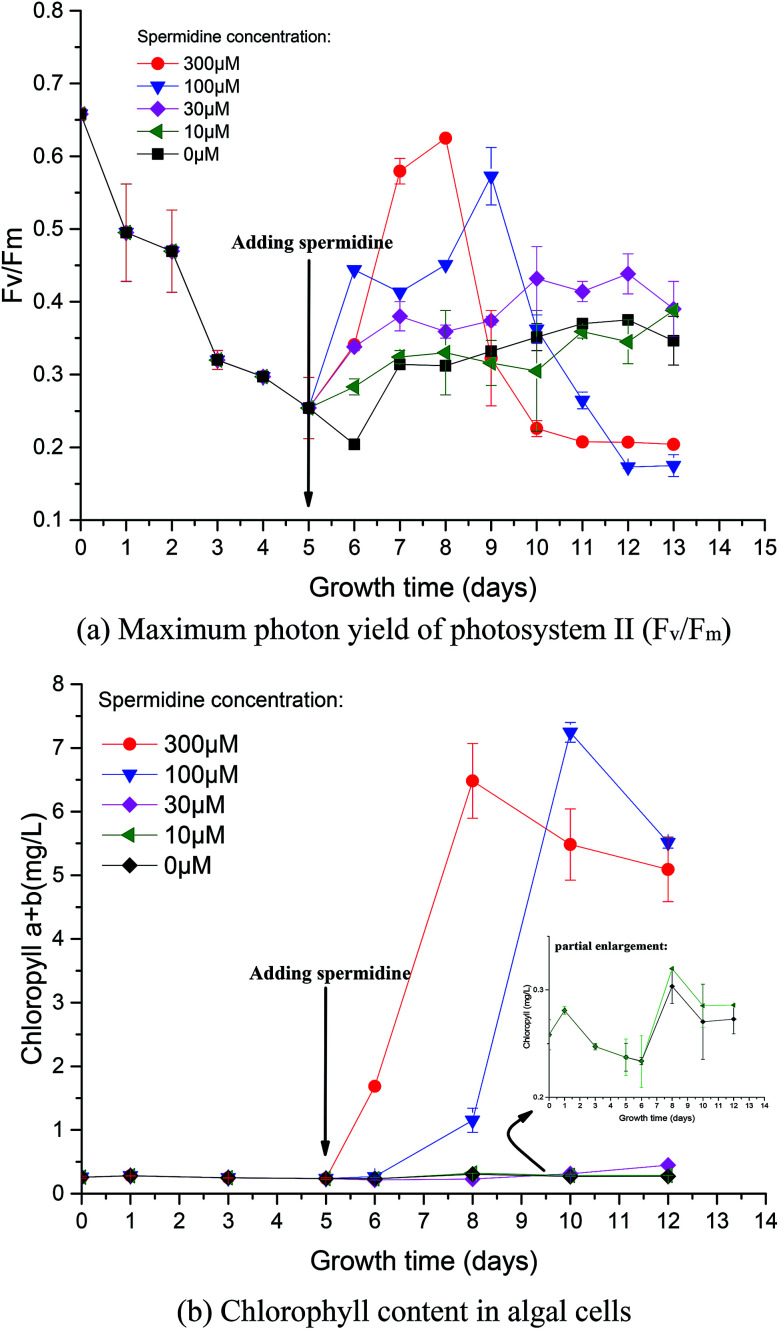
Photosynthesis (a) and chlorophyll synthesis (b) of *Chlorella* under a high CO_2_ concentration (15%) and high light intensity (30 000 lux).

During phase I, microalgal growth was significantly inhibited, to almost stationary (Fig. 1a). This was because the pH of the medium rapidly decreased due to the continuous introduction of a high CO_2_ concentration and high light intensity that directly caused photoinhibition of the microalgae. The *F*_v_/*F*_m_ of *Chlorella* decreased significantly during phase I (Fig. 2a), indicating that the photosynthetic apparatus in the microalgal cells was partially damaged, which resulted in a significantly decreased utilization efficiency of light energy. Thus, the excessive light energy could not be utilized by microalgal cells and ROS was subsequently produced in the photosynthetic apparatus to inhibit cell photosynthesis. During phase II (days 6–14), treatments were subjected to different spermidine concentrations (0, 10, 30, 100, 200, 300 and 400 μM). At the high CO_2_ concentration and high light intensity, almost no growth promotion was observed at extremely low concentrations of spermidine (10 and 30 μM), which was different than that under air culture conditions.^8^ It was speculated that adding extremely low concentrations of spermidine would not sufficiently enhance the stress resistance of *Chlorella* to overcome the inhibitory effects of high CO_2_ concentrations and high light intensity on microalgal growth.

Similarly, in order to compare the effects of spermidine and NaOH on the growth of *Chlorella*, a 100 μM NaOH treatment was set up. On the first day after the addition, the pH of the 100 μM NaOH and 100 μM spermidine treatments were basically the same, indicating that simply increasing the pH did not promote microalgal growth. This was due to the fact that in addition to the inhibitory effect of low pH, the high light intensity caused photoinhibition of *Chlorella*, which together hindered *Chlorella* growth. The growth rates in the 100, 200 and 300 μM spermidine treatments were significantly improved (Fig. 1a). The inhibitory effect of the high CO_2_ concentration and high light intensity was counteracted with a low concentration of *Chlorella*. In the 100, 200 and 300 μM spermidine treatments, the biomass production of *Chlorella* gradually increased with increasing spermidine concentrations. This indicates that higher concentrations, within the tested range, results in a stronger spermidine effect on the stress resistance and growth promotion of *Chlorella*. The addition of exogenous spermidine enhanced the expression of key enzymes of photosystem I (PSI) and PSII, ATP synthase and transportase in the algal cells and enhanced the activity of antioxidant enzymes, such as SOD, electron transport and energy supply. These changes contributed to decreased oxidative stress damage to cells, as well as enhanced intracellular chlorophyll synthesis and photosynthesis. The combined effects ultimately led to a significant increase in biomass production in most treatments, with the exception of the 400 μM spermidine treatment, which decreased significantly. The excessive concentration of spermidine in the 400 μM treatment produced a toxic effect on microalgal growth, resulting in a 16.96% decrease in biomass yield when compared with the 300 μM treatment. Excessive exogenous spermidine disrupted the existing intracellular metabolic balance and pH equilibrium, and its interaction with RNA/DNA inhibited normal cellular differentiation.

### 3.2. Photosynthesis and synthesis of chlorophyll in cells

Photosynthetic activity in microalgae is represented by *F*_v_/*F*_m_.^20^ The *F*_v_/*F*_m_ value has been found to be relatively constant in non-stressed cultures, while it is lower in stressed cultures.^22^ Mallick and Mohn^23^ studied the chlorophyll fluorescence of the green microalga *Scenedesmus* under different metal stresses and concluded that the *F*_o_/*F*_v_ ratio could be used as a powerful tool in metal-stress research. In this study, *F*_v_/*F*_m_ values were used to characterize damage to *Chlorella* photosynthesis and the photosynthetic system. *F*_v/_*F*_m_ represents the maximum quantum yield of PSII, reflecting the potential maximum photosynthetic capacity. The value of *F*_v_/*F*_m_ is often used to reflect the extent stress on microalgae.

The *F*_v_/*F*_m_ value at the beginning of microalgal cultivation was 0.658 and it decreased continuously during phase I, reaching the minimum value on day 6 (Fig. 2a), which was 69.00% lower than the initial value. When the microalgae were subjected to high light intensity, the excess electrons in the photosynthetic electron transport chain induced the generation of numerous ROS, thereby causing photosynthesis inhibition, pigment co-oxidation, lipid peroxidation, membrane destruction and protein denaturation. The photosynthetic structure was damaged under the low pH and high light intensity; therefore, the microalgal cells could not effectively utilize light energy. After adding exogenous spermidine, the *F*_v_/*F*_m_ values of the 30, 100 and 300 μM treatments were significantly improved, indicating that these spermidine concentrations had a repairing effect on the microalgal photosynthetic system, thereby enabling the microalgal cells to utilize light energy normally. However, there was no notable difference between the 10 μM treatment and the control.

The changes in chlorophyll content were also investigated. After spermidine addition, the chlorophyll content of the 300 and 100 μM treatments increased significantly, with peaks observed on the third and fifth days, respectively. The chlorophyll synthesis rate then became slow and long-term exposure to high light intensity triggered serious damage to the microalgal cells as the decomposition of chloroplast^24^ resulted in a lower chlorophyll content after the peaks. It could be observed that the 100 μM treatment had a significant lag in the improvement of microalgal stress resistance, indicating that there was a significant difference in the alleviation effect along the treatment gradient. Lower concentrations of spermidine required a longer time to improve the stress resistance of *Chlorella* cells. Furthermore, there was no obvious difference between the 10 and 30 μM treatments and the control, indicating that extremely low concentrations of exogenous spermidine did not enhance the stress resistance of *Chlorella* cells to high CO_2_ and high light intensity.

It is noteworthy that the change in chlorophyll content per unit cell and the change in *F*_v_/*F*_m_ values were basically consistent with the specific growth rate of the cells (Fig. 2 and 1b). It was speculated that chlorophyll synthesis and the higher photosynthetic efficiency were the intrinsic drivers of spermidine-enhanced stress resistance and increased cell growth rate under the high CO_2_ concentration and high light intensity.

**Fig. 3:**
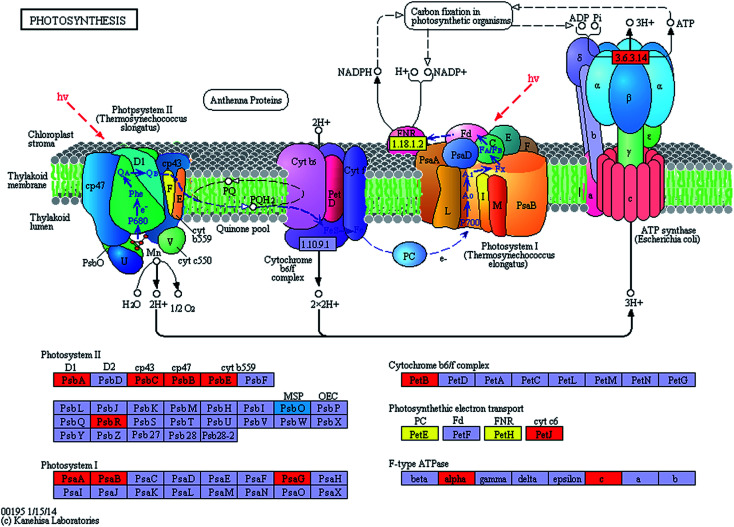
Effects of spermidine on gene transcript expression in the photosynthetic system of *Chlorella* under a high CO_2_ concentration (15%) and high light intensity (30 000 lux). Red represents up-regulation, blue represents down-regulation, yellow represents both up-regulation and down-regulation and others represent no change.

The expression levels of related enzymes in the photosynthetic system of the spermidine treatments and the control under high light intensity and high CO_2_ were also investigated. Exogenous spermidine increased the expression of most of the key enzymes in the photosynthetic apparatus (Fig. 3). The difference in transcript abundance between the spermidine treatment and the control was highest for ATP synthase (EC: 3.6.3.14) with an increase of log_2_ FC 3.2536. This enzyme promotes ATP generation for the carbon fixation process, thus accelerating CO_2_ fixation. The transcript abundances of H^+^-ATPases including ATPF1A and ATPF0C (F-type ATPases) and ATPeV0A (V-type ATPases) in the spermidine treatments were up-regulated by times of log_2_ FC 3.2151, log_2_ FC 3.2536 and log_2_ FC 4.7389, respectively. On the one hand, the up-regulation of ATP synthase expression helped to generate more energy to resist the adverse external environment. On the other hand, consumption of surplus ATP (generated as a result of cyclic electron transport by ATPase) helped the microalgal cells efficiently control intracellular pH, which might be achieved by pumping protons from the cytoplasm into vacuoles.^25^ At the same time, during the active growth process *via* nitrate uptake, the increased pH of the culture solution gradually helped the cells to grow better. The expression levels of related PSI and PSII enzymes also increased significantly (Table 1), which enhanced electron transfer in the microalgal photosynthetic systems. The higher cyclic electron transport of PSI generated the additional ATP necessary for support of pH homeostasis in the algal cells, thus helping the cells to tolerate high CO_2_ concentrations^26^ and to alleviate the oxidative stress effects of photoinhibition caused by high light intensity. It is noteworthy that the expression of psbO (PSII oxygen-evolving enhancer protein 1) was down-regulated, with a log_2_ FC −4.5043 difference in transcript abundance between the spermidine treatment and control. It was speculated that under photoinhibition conditions, a large amount of ROS accumulated in the cells and that the lower psbO expression could effectively reduce oxygen production in the photosynthetic apparatus and decrease damage to the cells.

**Table 1:** Annotation of significantly different unigenes related to *Chlorella* photosynthesis after spermidine treatment under a high CO_2_ concentration (15%) and high light intensity (30 000 lux)^a^

KO no.	EC no.	Name	Definition	log_2_ FC
K02703	1.10.3.9	psbA	Photosystem II P680 reaction center D1 protein	3.2796
K02705		psbC	Photosystem II CP43 chlorophyll apoprotein	3.1587
K02704		psbB	Photosystem II CP47 chlorophyll apoprotein	3.0463
K02707		psbE	Photosystem II cytochrome b559 subunit alpha	2.1759
K02716		psbO	Photosystem II oxygen-evolving enhancer protein 1	−4.5043
K03541		psbR	Photosystem II 10 kDa protein	5.1544
K02689		psaA	Photosystem I P700 chlorophyll a apoprotein A1	3.3221
K02690		psaB	Photosystem I P700 chlorophyll a apoprotein A2	3.6505
K08905		psaG	Photosystem I subunit V	2.5495

a log_2_ FC is the log base 2 of the difference in transcript abundance between the spermidine treatment and control.

### 3.3. Microstructure of *Chlorella* cells

**Fig. 4:**
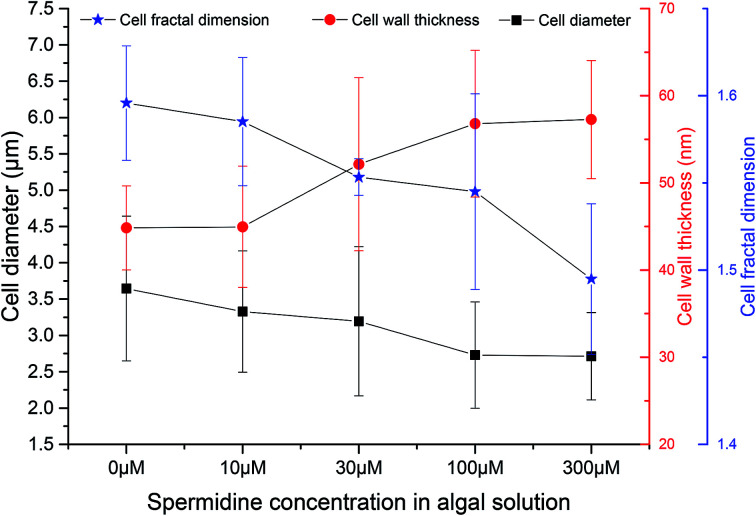
Effects of spermidine on the cellular microstructure of *Chlorella* under a high CO_2_ concentration (15%) and high light intensity (30 000 lux).

TEM and SEM observations were performed on samples from the 0, 10, 30, 100 and 300 μM treatments on day 10 (five days after exposure to exogenous spermidine) to explore the effects of different spermidine concentrations on the microstructure and surface morphology of microalgal cells. The average diameter of *Chlorella* decreased with increasing spermidine concentrations (Fig. 4). Compared with the control, the average diameter of *Chlorella* in the 10, 30, 100 and 300 μM treatments successively decreased by 8.70%, 12.35%, 25.15% and 25.54%, respectively. This was because exogenous spermidine promoted cell division and proliferation, the effect of which strengthened with increasing concentrations, leading to a higher proportion of small cells and a lower average cell diameter.

At the same time, the cell wall thickened with increasing spermidine concentrations. Compared with the control, the thickness of the 10, 30, 100 and 300 μM treatments increased by 0.28%, 16.29%, 26.68%, and 27.73%, respectively. Moreover, the 10 μM treatment showed almost no influence on cell wall thickness. It was speculated that higher concentrations of spermidine (30, 100 and 300 μM) could promote the synthesis of cell protoplasts and cell wall components, thereby providing the cells with a formidable defense against the environment. Thus, the cells were protected against damage to their internal structure under low pH and high light intensity, helping them grow normally under the adverse conditions.

Mandelbrot^27^ first proposed the concept of fractal geometry. Fractal dimension reflects the effectiveness of space occupation by complex objects, and it is a parameter of the irregular degree of complex objects. In order to investigate the effects of different spermidine concentrations on the microscopic structure of the *Chlorella* cells, the concept of fractal dimension was used to quantitatively study the surface morphology of cells treated with different spermidine concentrations. Compared with the control, the fractal dimension of the 10, 30, 100 and 300 μM treatments decreased by 0.67%, 2.67%, 3.19% and 6.33%, respectively. Under 15% CO_2_ and high light intensity, the surface of the microalgal cells was damaged and the cell membrane was rough. Exogenous spermidine lightly contributed to smoothing the corrugation and deformation degree of the cells. Therefore, it was speculated that exogenous spermidine increased the cell wall thickness of the microalgae while smoothing the cell surface by repairing parts of the damage. The treated cells resisted H^+^ damage as the thickening of their cell wall helped to adapt to the low pH.

### 3.4. Intracellular antioxidant enzyme activity in *Chlorella*

**Fig. 5:**
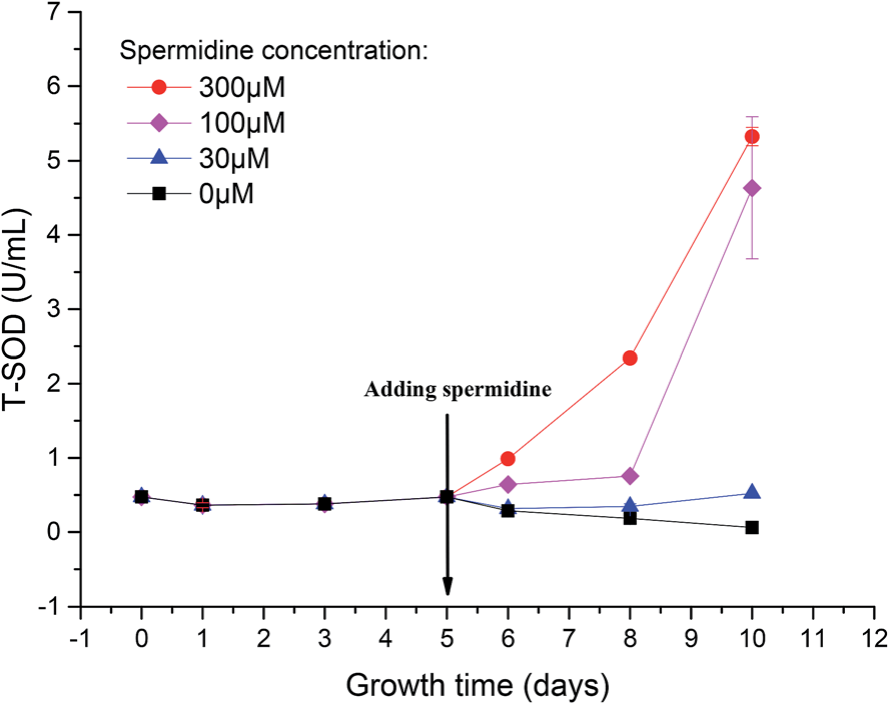
Enhanced activity of total antioxidant enzymes in *Chlorella* cells with spermidine under a high CO_2_ concentration (15%) and high light intensity (30 000 lux).

To reveal the extent of microalgal resistance against oxidative stress induced by high light intensity, the activity of ROS scavenging enzymes was measured. One of the major roles of polyamines in the cell is to provide resistance to intracellular and environmental stress, including that caused by ROS, temperature changes, osmotic pressure or other toxic compounds.^1^ The activity of T-SOD in the cells of the control decreased by 87.23%, from the initial 0.47 U mL^−1^ to 0.06 U mL^−1^ (Fig. 5). This indicates that the activity of the intracellular antioxidant enzymes was inhibited under the continuous influence of 15% CO_2_ and high light intensity. The oxidative stress triggered by low pH and photoinhibition of cells further damaged the cell structure and destroyed the normal growth and metabolic pathways. After treatment with spermidine, the intracellular T-SOD activity improved compared to the control. The T-SOD activity in the 100 and 300 μM treatments increased by 34.92% and 107.94%, respectively, compared with day 5, and steadily increased thereafter. The lowest concentration of spermidine (10 μM) was not enough to compensate for the oxidative stress damage caused by the high light intensity and high CO_2_ concentration so the intracellular T-SOD activity continued to decrease. On day 10 of the experiment, the activity of the 30 μM treatment increased by 10.43% compared with the initial T-SOD activity, while the activities of the 100 and 300 μM treatments were 8.77 and 10.23 times higher, respectively. The exogenous spermidine significantly enhanced the intracellular T-SOD activity, which helped to alleviate the oxidative stress caused by the high light intensity and high CO_2_ concentration, thus enhancing the stress resistance of the *Chlorella* cells. The high CO_2_ concentration and high light intensity gave rise to oxidative stress in the cells. Excess ROS triggered damaging effects to the membrane system and finally inhibited growth of the cells. SOD, an important antioxidative enzyme that uses free radicals as substrates, can convert O^2−^ to H_2_O_2_ and O_2_, thereby preventing the generation of superoxide anion radicals.^28^ Spermidine is one of the polyamines involved in the regulation of a substantial number of metabolic reactions that are presumably related to the survival of plants in stressful environments.^29^ Spermidine can enhance the activity of antioxidant enzymes to decrease the intensity of oxidative stress.^30,31^ In this case, it mainly enhanced antioxidant activity, which effectively removed free radicals in cells. Therefore, oxidative damage was reduced and resistance to high light intensity and high CO_2_ concentrations were promoted, which allowed cells to return to normal growth.

When cells experienced photoinhibition, O_2_ acted as an oxidant to accept electrons from the PSI reducing side to form O^2−^, which is toxic to cells. The increased T-SOD activity helped the cells to maintain a certain level of photosynthetic electron flow while eliminating this toxic effect, thereby reducing the toxicity of excess light energy to the photosynthetic system.^32^ As a result, light suppression was alleviated and the *F*_v_/*F*_m_ value subsequently increased. Moreover, enhancement of antioxidant enzyme activity was beneficial for repairing the damage caused by ROS.

## 4. Conclusions

Exogenous spermidine (100–300 μM) enhanced the resistance of *Chlorella* to a high CO_2_ concentration and high light intensity and increased the biomass yield from 0.11 g L^−1^ (0 μM) to 1.71 g L^−1^ (300 μM). Compared with the 0 μM group the fractal dimension of the 300 μM treatment decreased by 6.33%. Additionally, the accelerated cell division decreased the cell diameter from 3.64 μm to 2.71 μm and the activity of total superoxide dismutase (T-SOD) increased from 0.48 U mL^−1^ to 5.33 U mL^−1^. Finally, no obvious promotion effect was observed when the spermidine concentration was extremely low (≤30 μM).

## Conflicts of interest

There are no conflicts to declare.

## Supplementary Material

## References

[cit1] Gevrekci A. O. (2017). World J. Microbiol. Biotechnol..

[cit2] Duan J., Li J., Guo S., Kang Y. (2008). J. Plant Physiol..

[cit3] Zhang Y., Zhang H., Zou Z., Liu Y., Hu X. (2015). Phytochemistry.

[cit4] Lou Y., Guan R., Sun M., Han F., He W., Wang H., Song F., Cui X., Zhuge Y. (2018). Ecotoxicology.

[cit5] Murkowski A. (2001). Biol. Plant..

[cit6] Yin L., Wang S., Tanaka K., Fujihara S., Itai A., Den X., Zhang S. (2016). Plant, Cell Environ..

[cit7] Li L., Gu W., Li J., Li C., Xie T., Qu D., Meng Y., Li C., Wei S. (2018). Plant Physiol. Biochem..

[cit8] Shaw S., Abate M., Du X., Ying Y. (2018). Pak. J. Bot..

[cit9] Czerpak R., Bajguz A., Piotrowska A., Dobrogowska R., Matejczyk M., Wieslawski W. (2003). Acta Soc. Bot. Pol..

[cit10] Piotrowska-Niczyporuk A., Bajguz A., Zambrzycka E., Godlewska-Żyłkiewicz B. (2012). Plant Physiol. Biochem..

[cit11] Kim S., Jin Y., Choi I., Park Y., Seo J. (2015). Metab. Eng..

[cit12] Cheng D., Li X., Yuan Y., Yang C., Tang T., Zhao Q., Sun Y. (2019). Sci. Total Environ..

[cit13] García-Cubero R., Moreno-Fernández J., García-González M. (2018). Waste Biomass Valorization.

[cit14] Cheng J., Yang Z., Huang Y., Huang L., Hu L., Xu D., Zhou J., Cen K. (2015). Bioresour. Technol..

[cit15] Kumar K., Mishra S. K., Shrivastav A., Park M. S., Yang J. (2015). Renewable Sustainable Energy Rev..

[cit16] Ioannidis N. E., Kotzabasis K. (2007). Biochim. Biophys. Acta, Bioenerg..

[cit17] Unal D., Tuney I., Sukatar A. (2008). J. Photochem. Photobiol., B.

[cit18] Cheng J., Lu H., Huang Y., Li K., Huang R., Zhou J., Cen K. (2016). Bioresour. Technol..

[cit19] Porra R. J., Thompson W. A., Kriedemann P. E. (1989). Biochim. Biophys. Acta, Bioenerg..

[cit20] Parkhill J. P., Maillet G., Cullen J. J. (2001). J. Phycol..

[cit21] Pei M., Niu J., Li C., Cao F., Quan S. (2016). BMC Genomics.

[cit22] White S., Anandraj A., Bux F. (2011). Bioresour. Technol..

[cit23] Mallick N., Mohn F. H. (2003). Ecotoxicol. Environ. Saf..

[cit24] He Q., Yang H., Wu L., Hu C. (2015). Bioresour. Technol..

[cit25] Gogarten J. P., Taiz L. (1992). Photosynth. Res..

[cit26] Miyachi S., Iwasaki I., Shiraiwa Y. (2003). Photosynth. Res..

[cit27] Mandelbrot B. B. (1975). J. Fluid Mech..

[cit28] Tripathi B. N., Mehta S. K., Amar A., Gaur J. P. (2006). Chemosphere.

[cit29] Sung M., Chow T., Lee T. (2011). J. Phycol..

[cit30] Kuehn G. D., Phillips G. C. (2005). Crit. Rev. Plant Sci..

[cit31] Valdes-Santiago L., Ruiz-Herrera J. (2014). Front. Chem..

[cit32] Demmig-Adams B. (1998). Plant Cell Physiol..

